# The efficacy of reduced-visit prenatal care model during the coronavirus disease 2019 pandemic: A protocol for systematic review and meta-analysis

**DOI:** 10.1097/MD.0000000000025435

**Published:** 2021-04-16

**Authors:** Xiaoli Wang, Ying Wang, Lin Liang

**Affiliations:** a Department of Obstetrics, Maternal and Child Health Hospital of Hubei Province, Tongji Medical College, Hua Zhong University of Science and Technology; b Department of Obstetrics, General Hospital of the Central Theater Command of the Chinese People's Liberation Army, Wuhan, Hubei, China.

**Keywords:** COVID-19, meta-analysis, protocol, reduced-visit prenatal care, review

## Abstract

**Background:**

While this reduced-visit prenatal care model during the COVID-19 pandemic is well-intentioned, there is still a lack of relevant evidence to prove its effectiveness. Therefore, in order to provide new evidence-based medical evidence for clinical treatment, we undertook a systematic review and meta-analysis to assess the efficacy of reduced-visit prenatal care model during the COVID-19 pandemic.

**Methods:**

The online literature will be searched using the following combination of medical subject heading terms: “prenatal care” OR “prenatal nursing” AND “reduced-visit” OR “reduce visit” OR “virtual visit.” MEDLINE, EMBASE, Cochrane Central Register of Controlled Trials, and Web of Science will be searched without any language restrictions. A standard data extraction form is used independently by 2 reviewers to retrieve the relevant data from the articles. The outcome measures are as following: pregnancy-related stress, satisfaction with care, quality of care. The present study will be performed by Review Manager Software (RevMan Version 5.3, The Cochrane Collaboration, Copenhagen, Denmark). *P* < .05 is set as the significance level.

**Results:**

It is hypothesized that reduced-visit prenatal care model will provide similar outcomes compared with traditional care model.

**Conclusions:**

The results of our review will be reported strictly following the Preferred Reporting Items for Systematic Reviews and Meta-Analyses (PRISMA) criteria and the review will add to the existing literature by showing compelling evidence and improved guidance in clinic settings.

**OSF registration number:**

10.17605/OSF.IO/WYMB7.

## 1 Introduction

Prenatal care is an important preventive health service used in developed countries around the world, which aims to reduce the risk of adverse pregnancy and childbirth by providing pregnant women with regular health assessments and information on pregnancy, delivery, birth, and parenthood. Most prenatal care occurs during routine office visits.^[1,2]^ In the United States, the American College of Obstetricians and Gynecologists recommends a uniform prenatal schedule that includes approximately 14 visits: every 4 weeks to 28 to 32 weeks of gestation, then every 2 weeks to 36 weeks, and finally once a week until delivery.^[3]^ This rhythm of care is codified largely based on the tradition of providing information for care plans designed to detect risks, such as hypertensive disorders in pregnancy. However, research does not necessarily support high numbers of visits for the majority of low-risk pregnancies. Indeed, there is a wide variation in visit schedules across countries, and higher numbers of visits do not necessarily correspond with better outcomes.^[4]^ While there is no doubt that antenatal care is important for maternal and child health, there is little evidence of a contact–response relationship between visit frequency and outcome.^[5]^

During the 2019 coronavirus disease (COVID-19) pandemic, the United States experienced the biggest transformation in prenatal care services in nearly a century—rapidly implementing multiple alternative prenatal care approaches to ensure access to prenatal services while maintaining social isolation, including reducing frequency and virtual visits.^[5–7]^ The COVID-19 pandemic provides a unique opportunity to evaluate reduced access plans and virtual access in real-world environments. The early detection of alternatives to early prenatal care is critical to help hospitals and health systems make decisions on how to maintain or modify prenatal care adaptations related to COVID-19 in response to the pandemic.^[8,9]^

While this reduced-visit prenatal care model during the COVID-19 pandemic is well-intentioned, there is still a lack of relevant evidence to prove its effectiveness. Therefore, in order to provide new evidence-based medical evidence for clinical treatment, we undertook a systematic review and meta-analysis to assess the efficacy of reduced-visit prenatal care model during the COVID-19 pandemic. It is hypothesized that reduced-visit prenatal care model will provide similar outcomes compared with traditional care model.

## 2 Materials and methods

### 2.1 Study protocol registration

The protocol was written following the Preferred Reporting Items for Systematic Reviews and Meta-Analyses Protocols (PRISMA-P) statement guidelines. The prospective registration has been approved by the Open Science Framework registries and the registration number is 10.17605/OSF.IO/WYMB7. Ethical approval and patient consent are not required because this study is a literature-based study.

### 2.2 Searching strategy

**Figure 1:**
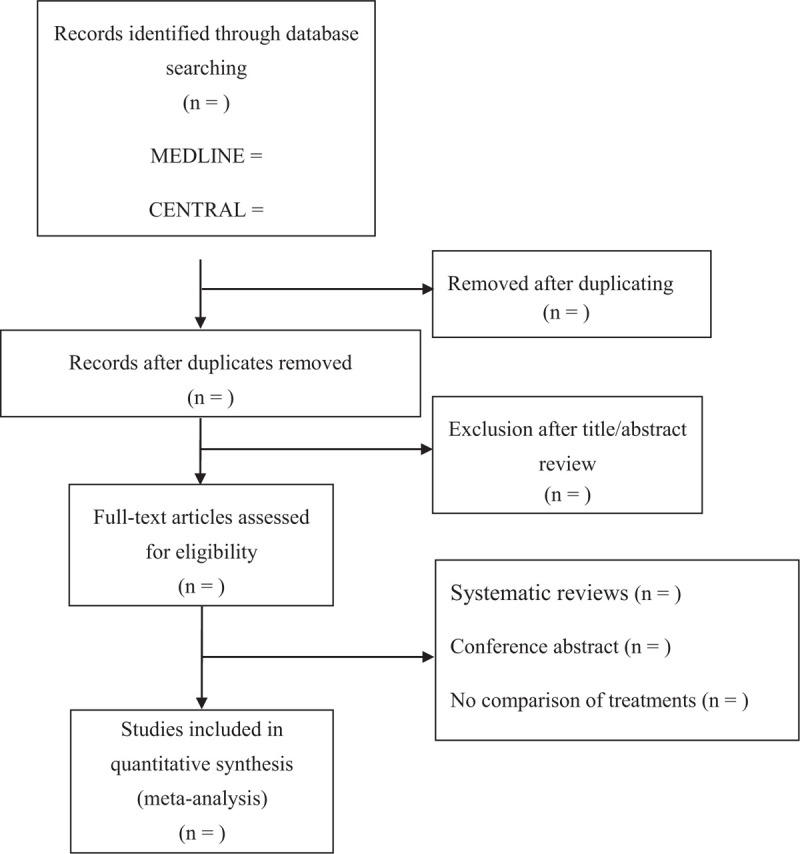
Flow diagram of study identification.

The online literature will be searched using the following combination of medical subject heading terms: “prenatal care” OR “prenatal nursing” AND “reduced-visit” OR “reduce visit” OR “virtual visit.” MEDLINE (PubMed), EMBASE (OVID), Cochrane Central Register of Controlled Trials (CENTRAL), and Web of Science (ISI database) will be searched without any language restrictions (Fig. 1). The reference lists of the included studies will be also checked for additional studies that are not identified with the database search.

### 2.3 Eligibility criteria

Study included in this review has to meet all of the following inclusion criteria in the PICOS order:

(1) population: pregnant women;
(2) intervention group (group 1): reduced-visit prenatal care model;
(3) comparison group (group 2): traditional prenatal care model;
(4) outcome measures: at least one of the following outcome measures was reported: pregnancy-related stress, satisfaction with care, quality of care;
(5) study design: randomized controlled trial or observational study.

Biomechanical studies, in vitro studies, review articles, techniques, case reports, letters to the editor, and editorials are excluded.

### 2.4 Data extraction

A standard data extraction form is used independently by 2 reviewers to retrieve the relevant data from the articles. These variables include author, study design, sample size, publishing date, population, type of interventions, type of controls, follow-up, and outcomes. The outcome measures are as following: pregnancy-related stress, satisfaction with care, quality of care. Data extraction is performed independently, and any conflict is resolved before final analysis. If data (e.g., SDs and SEs) are not presented in the original article, corresponding authors will be contacted to acquire the missing data. Otherwise, the results are extracted manually from the published figures. If necessary, we will abandon the extraction of incomplete data.

### 2.5 Data analysis

The present study will be performed by Review Manager Software (RevMan Version 5.3, The Cochrane Collaboration, Copenhagen, Denmark). Mean differences with a 95% confidence interval are assessed for continuous outcomes. *P* < .05 is set as the significance level. We examine heterogeneity graphically using forest plots and statistically by calculating the *I*^2^ statistic, which describes the percentage of the variability in effect estimates that is due to heterogeneity rather than sampling error (chance). We consider an *I*^2^ statistic >50% to be substantially heterogeneous. All outcomes are pooled on random-effect model. The Z test is used to assess the overall effect. A meta-analysis is conducted when ≥4 trials reported an outcome of interest. Subgroup analyses are planned by different follow-up periods. We also conduct the sensitivity analysis to evaluate whether any single study has the weight to skew on the overall estimate and data. Begg funnel plot is used to assess publication bias. If publication bias exists, the Begg funnel plot is asymmetric.

### 2.6 Study quality assessment

The quality of randomized trials will be assessed by Cochrane risk of bias tool for randomized controlled trials and the Risk Of Bias In Non-Randomized Studies - of Interventions for non-randomized, observational studies. Each paper will be reviewed by 1 reviewer and verified by a second and disagreements will be resolved by discussion with a third reviewer. A meta-analysis will be conducted when ≥3 trials reported an outcome of interest. We also will perform the sensitivity analysis to evaluate whether the differences of study design had an impact on the overall estimate and data. Review Manager software (v 5.4; Cochrane Collaboration) will be conducted for statistical investigation and a funnel plot analysis will be drawn to assess the publication bias if there are >10 studies included.

## 3 Discussion

Prenatal care is one of the most common preventive healthcare services in the United States, used by almost 4 million women each year. The COVID-19 pandemic provides a unique opportunity to evaluate reduced access plans and virtual access in real-world environments. The early detection of alternatives to early prenatal care is critical to help hospitals and health systems make decisions on how to maintain or modify prenatal care adaptations related to COVID-19 in response to the pandemic. While this reduced-visit prenatal care model during the COVID-19 pandemic is well-intentioned, there is still a lack of relevant evidence to prove its effectiveness. Therefore, in order to provide new evidence-based medical evidence for clinical treatment, we undertook a systematic review and meta-analysis to assess the efficacy of reduced-visit prenatal care model during the COVID-19 pandemic. It is hypothesized that reduced-visit prenatal care model will provide similar outcomes compared with traditional care model.

## Author contributions

**Conceptualization:** Xiaoli Wang, Ying Wang.

**Data curation:** Ying Wang, Ying Wang.

**Formal analysis:** Xiaoli Wang, Ying Wang.

**Funding acquisition:** Lin Liang.

**Investigation:** Xiaoli Wang, Ying Wang.

**Methodology:** Xiaoli Wang, Ying Wang.

**Project administration:** Lin Liang.

**Resources:** Lin Liang.

**Software:** Xiaoli Wang, Ying Wang.

**Supervision:** Lin Liang.

**Validation:** Lin Liang.

**Visualization:** Ying Wang.

**Writing – original draft:** Xiaoli Wang, Ying Wang.

**Writing – review & editing:** Lin Liang.
